# Impact of non-surgical periodontal therapy on serum Resistin and periodontal pathogen in periodontitis patients with obesity

**DOI:** 10.1186/s12903-020-1039-3

**Published:** 2020-02-14

**Authors:** Khairunnisa Md Tahir, Ainul Haliza Ab Malek, Rathna Devi Vaithilingam, Roslan Saub, Syarida Hasnur Safii, Mohammad Tariqur Rahman, Fathilah Abdul Razak, Aied M Alabsi, Nor Adinar Baharuddin

**Affiliations:** 1 Department of Restorative Dentistry, Faculty of Dentistry, University of Malaya, Lembah Pantai, 50603 Kuala Lumpur, Malaysia; 2 Department of Community Oral Health & Clinical Prevention, Faculty of Dentistry, University of Malaya, Lembah Pantai, 50603 Kuala Lumpur, Malaysia; 3 Dean Office, Faculty of Dentistry, University of Malaya, Lembah Pantai, 50603 Kuala Lumpur, Malaysia; 4 Department of Oral & Craniofacial Sciences, Faculty of Dentistry, University of Malaya, Lembah Pantai, 50603 Kuala Lumpur, Malaysia; 5 Faculty of Dental Medicine, Universitas Airlangga, Surabaya, Indonesia; 6 Faculty of Dentistry, MAHSA University, Jalan SP2, Bandar Saujana Putra, 42610 Jenjarom Kuala Langat, Selangor Malaysia

**Keywords:** Obese, Periodontitis, Cytokine, Periodontal pathogens, Periodontal therapy

## Abstract

**Background:**

Non-surgical periodontal therapy (NSPT) known as gold standard treatment in managing periodontitis. The aim of this study was to investigate the response of NSPT in periodontitis subjects who were obese. Clinical parameters of periodontitis, changes in serum resistin and periodontal pathogens in subgingival plaque were compared before and after NSPT in periodontitis subjects who were obese and with normal weight.

**Methods:**

A total of 48 periodontitis subjects (obese, *n* = 18; normal weight, *n* = 30) were recruited (hereafter will be referred as participants) to participate into a prospective, before and after clinical trial. Obesity status is defined by body mass index (BMI) criteria (obese: ≥30 kg/ m^2^; normal weight < 25 kg/m^2^). Visible Plaque Index (VPI), Gingival Bleeding Index (GBI), Probing Pocket Depth (PPD) and Clinical Attachment Loss (CAL) were recorded; and serum and plaque were collected at baseline and following 12 weeks post-NSPT. Serum resistin level was analyzed using enzyme-linked immune-sorbant assay (ELISA), while detection of periodontal pathogens in dental plaque were carried out using real time PCR (qPCR).

**Results:**

Following NSPT, means VPI and GBI showed significant improvement between obese and normal weight groups (*p* <  0.05), but no difference in means PPD and CAL was observed between groups. Obesity remained as a predictor for VPI and GBI after adjusting for smoking habit. No significant difference was observed in serum resistin level and mean counts for *P. gingivalis, T. forsythia*, and *P. intermedia* between obese and normal weight groups following NSPT.

**Conclusions:**

Regardless of obesity status, NSPT has a significant impact on VPI and GBI in periodontitis subjects. However, the impact of NSPT towards serum resistin and periodontal pathogens was non-significant in those with periodontitis.

**Trial registration:**

This study followed the Consolidation Standards of Reporting Trials Statement and retrospectively registered on 26/11/2015 at clinicaltrials.gov (No. NCT02618486).

## Background

Periodontal therapy is aimed to provide functional dentition and comfort for patients [1] with periodontitis. Non–surgical periodontal therapy (NSPT) focuses on elimination of bacterial plaque on the root surface by means of scaling and root surface debridement and preparing the root surface for healing. NSPT has been shown to provide improvement in clinical parameters namely, Visible Plaque Index (VPI), Gingival Bleeding Index (GBI), Probing Pocket Depth (PPD), Clinical Attachment Level (CAL), as well as reduction inflammation and in periodontal pathogens. NSPT was shown to induce a shift from a pre-dominant Gram negative to a Gram positive subgingival microbiota in the general population [2] and to significantly reduce total bacteria counts and positive sites of *P. gingivalis* and *T. forsythia* [3, 4]. NSPT is also expected to reduce the serum resistin level, which otherwise is present in higher amount in case of periodontal inflammation with periodontitis patients as compared to healthy individuals [5].

The outcome of the NSPT might vary depending on the severity of the disease as well as the other health complications of the patients such as obesity [6, 7]. Previous research showed that mean serum resistin was higher in obese subjects with periodontitis followed by non-obese periodontitis and non-obese healthy [8]. Following 2 months post-NSPT, greater reduction in resistin was observed in normal weight with periodontitis than obese periodontitis subjects [9]. In contrast, previous studies which compared obese and non-obese subjects with periodontitis following NSPT showed no significant difference in terms of serum levels of resistin at baseline and 3 months post-NSPT [5, 10]. Similarly, obesity condition could enhance the risk of patients to exhibit periodontitis by having high numbers of pathogenic subgingival species. Previous studies demonstrated that mean counts of *P. gingivalis* and *T. forsythia* were higher in obese with periodontitis [11, 12].

Resistin is a 12.5 kDA cysteine-rich secretory protein with mature sequence consists of 108 amino acids. Resistin has been expressed in macrophages, neutrophils and lymphocytes, and play various regulatory roles in biological processes including inflammation [13]. The role of resistin in the inflammatory pathway has been suggested through a nuclear factor NF-κB pathway [14]. Systematic and meta-analysis study reported high level of serum resistin observed in periodontitis patients as compared to healthy individuals [15]. This could be explained by the increased local pro-inflammatory cytokine levels in patients with periodontal inflammation, which is also associated with a state of elevated localized inflammatory burden. On the same note, obese subjects also showed elevated levels of serum inflammatory biomarkers (secreted from adipocytes), which modulate inflammatory responses indicating its possible inflammatory role in periodontitis.

The lack of evidence in this area and conflicting outcomes from previous studies warrant further investigations. More specifically, studies on the potential outcome of NSPT on the periodontal parameters, serum resistin level, and periodontal pathogens counts in periodontitis with obesity remains largely scanty. Thus, it remains unclear whether NSPT has a significant impact on these obese patients and normal weight patients, with periodontitis. Therefore, this study was aimed to evaluate the impact of NSPT on clinical parameters, serum resistin level and periodontal pathogen count in periodontitis patients with obesity and with normal weight.

## Methods

### Experimental design and ethics approval

This was a prospective study, before and after clinical trials, conducted at the Faculty of Dentistry, University of Malaya between March 2013 and October 2015. Ethical approval was obtained from the Medical Ethics Committee Faculty of Dentistry, University of Malaya (DF PE1501/0085(L)).

### Participants

The subjects were recruited from those who came for periodontal treatment at the Faculty of Dentistry, University of Malaya. Case definition for periodontitis by Eke et al. (2012) was used [16]. The inclusion criteria: (i) BMI for obese > 30.0 kg/m^2^ and normal weight < 25.0 kg/m^2^ with loosened clothing and no shoes [17] (ii) minimum number of teeth 12 (iii) age > 30 years. The exclusion criteria: (i) history of periodontal treatment in last six months, (ii) on medication namely antibiotics and topical/systemic steroid treatment in last four months, (iii) current or planning for pregnancy during the intervention period, (iv) lactating mothers, (iv) mentally handicapped, (v) history of valve replacement and rheumatic heart disease which require antibiotic coverage. Self-reported questionnaire was used to obtain information on sociodemographic, education status, medical history and habits. Radiograph was taken on case to case basis.

### Sample size calculation

The sample size calculation was determined based on detectable mean difference of 0.5 mm in CAL between 2 groups [18], a standard error of 5% (*p* = 0.05) and a power of 90% (0.9). The estimated sample size was 27 participants for each test and control groups. Due to anticipated 10% for dropouts which had been concluded from previous studies, thus a total of 30 participants per group were required. G*Power software version 3.1 was used [19] and *T*-test for independent means in an analysis statistical program was selected.

### Clinical assessment

The intra-examiner calibration involved an examiner (AH). The reproducibility of the clinical measurement was performed on 84 sites evaluated for PPD and CAL. The intra-examiner agreement (κ values) for PPD and CAL were 0.73 and 0.87 respectively. For inter-examiner calibration, 4 examiners were calibrated against an experienced periodontist on 168 sites for PPD and CAL. The inter-examiner agreement (κ values) for PPD and CAL were 0.79 and 0.87 respectively. Periodontal measurements and treatment was performed by the same examiner (AH). Full mouth Visible Plaque Index (VPI) [20], Gingival Bleeding Index [20], (VPI and GBI; absence or presence), probing pocket depth (PPD) and clinical attachment level (CAL) were assessed at six sites per tooth (mesiobuccal, midbuccal, distobuccal, mesiopalatal, midpalatal and distopalatal). For both groups, the measurements were carried out at baseline and at 12 weeks post-NSPT.

### Non-surgical periodontal treatment (NSPT)

All patients were given (i) standardized oral hygiene instruction namely tooth brushing with modified Bass technique and flossing, (ii) full mouth scaling; and (iii) root surface debridement at sites with PPD ≥ 5 mm [21] using an ultrasonic scaler Sirona C8 03653 (Dentsply Sirona, New York, USA) and Graceys curettes (Hu Friedy, Chicago, USA). The treated periodontal pockets were irrigated with 0.12% chlorhexidine gluconate (Oradex®). Patients were advised to rinse their mouth three times daily for 14 days using 15 ml of 0.12% chlorhexidine gluconate mouthwash [22]. The primary outcomes of the study were serum resistin levels and periodontal pathogens counts.

### Quantification of resistin

Levels of resistin was measured in serum from each participant at baseline (prior to NSPT) and at 12 weeks after NSPT using ELISA kits according to instructions of the manufacturer (Quantikine HS, R&D System Europe Ltd., UK). Briefly, 5 mL of venous blood from each participant was centrifuged at 1000 g for 15 min and stored in Eppendorf tubes at − 80 °C until further analysis. Thawed serum samples were diluted 25× using the diluent provided in the kit for ELISA. Purified resistin (100 ng/mL) was used as standard while the assay diluent was used as the blank. In a well of a 96 wells micro-titer plate, 100 μL of assay diluent with either 100 μL of diluted standard or diluted were added. Following 2-h incubation at room temperature, the wells were aspirated and washed for 3–4 times using the wash buffer. 200 μL of human conjugate (monoclonal antibody against resistin conjugated to horseradish peroxidase) was added to each well and incubated at room temperature for another 2-h. The washing steps were repeated. 200 μL of substrate solution (stabilized hydrogen peroxide and tetramethylbenzidine) was added to each well and incubated in dark for 30 min at room temperature. 50 μL of stop solution was added to the wells to stop the reaction. The absorbance was recorded at a wavelength of 450 nm. The concentration serum resistin level (ng/mL) was determined using the standard curve that had been running using the positive control. The minimum detection limits for resistin was 0.026 ng/mL as stated in the ELISA kit. The assay procedure was performed in duplicate.

### Detection and quantification of periodontal microorganisms using qPCR

Subgingival plaque samples were collected from the deepest periodontal sites using sterile curette from each participant at baseline (prior to NSPT) and at 12 weeks after NSPT. Prior to sampling, the tooth was isolated with a cotton roll and supragingival plaque was removed using cotton pellets. Following this, the subgingival plaque was taken using sterile Gracey’s curette by scrapping from the base of the pockets up to the gingival margin of at least four sites with the deepest probing depths which showed bleeding on probing [23]. The samples were placed in 1.5 ml Eppendorf tubes containing phosphate buffered saline (PBS) and stored at − 80 °C until assayed. The plaque samples were centrifuged at 13, 000 rpm for 5 min and the supernatant removed. Bacterial DNA was extracted using automated DNA extraction device (QIAcube, Qiagen Biotechnology, Kuala Lumpur, Malaysia) according to manufacturer’s instruction. The concentration and purity of all samples were measured with a spectrophotometer (NanoDrop 2000, Thermo Scientific, Wilmington, DE, USA).

A qPCR assay using singleplex TaqMan gene expression assays (Applied Biosystems®) was used for the detection and quantification of single-copy gene of W83 gene for *P. gingivalis*, ATCC 43037 gene for *T. forsythia*, and ATCC 25611 gene for *P. intermedia*. A 10-fold dilution series of genomic DNA at the concentrations 100 ng/ μL was used to create the standard curve as reference each periodontal pathogen. Each gene was duplicated for standard curve assays with negative control. The final graph with curve of correlation coefficient (R^2^) ≤ 0.99 and the efficiency ranges between 90 and 100% was used as the standard curve. Briefly, the reaction mixture contained 10 μL of 2× TaqMan Fast Advanced Master Mix (Applied Biosystems), 1 μL of 20 × gene expression assay, 7 μL nuclease-free water and 2 μL of template DNA from the plaque sample [23]. The assay was performed in a MicroAmp™ optical 96-well reaction plate (Applied Biosystems®). The quantification was performed using a thermal cycler (7500 Fast RT–PCR System, Applied Biosystems®) with a pre-defined programme; stages of enzyme activation at 95 °C for 20 s, 40 cycles of denaturation (at 95 °C for 3 s per cycle), annealing and extension phases (at 60 °C for 30 s each phase) [24].

### Statistical analysis

**Fig. 1:**
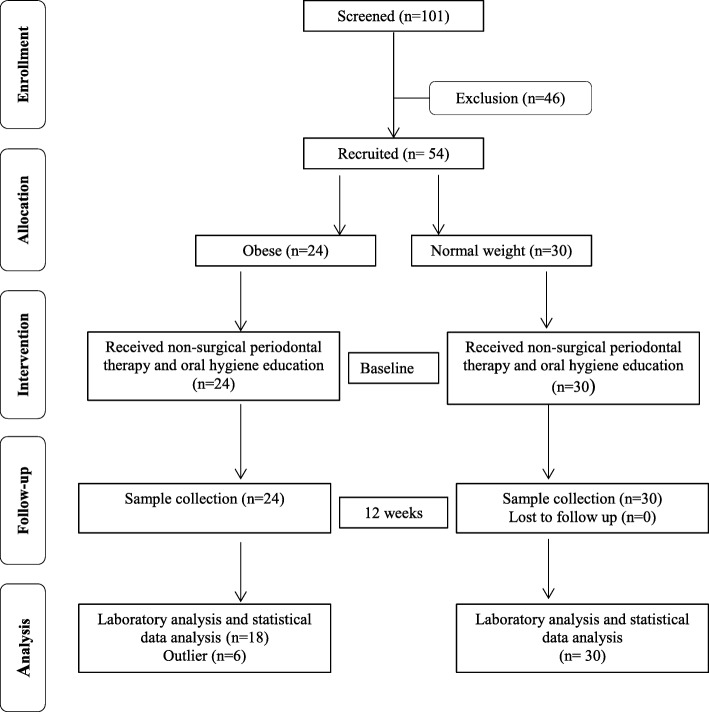
Study flow diagram

Data entry and statistical analysis were carried out using Statistical Package for the Social Sciences (SPSS), version 18.0 (SPSS Inc. Chicago, IL, USA). The data was tested for normality of distribution using Shapiro–Wilk test before testing for the hypothesis analysis. *P*–value of less than 0.05 was set as the statistically significant value. Independent *T*–test and Chi square test were used to analyze the socio-demographic profile of the participants. Comparison means between group at baseline and 12 weeks post NSPT were analyzed using Independent *T*-test or Mann-Whitney test. Repeated measures ANOVA was used to compare the changes (from baseline to 12 weeks post NSPT) in means of clinical parameters, serum resistin levels and periodontal pathogens within group and between groups. The significant level was determined when 95% CI showed no overlapping values. Regression analysis was employed to identify explanatory variables for obesity status, controlling the effects of other possible covariates. The flow of the study is shown in Fig. 1.

## Results

### Socio-demographic profile of the participants

Initially, there were 24 obese and 30 normal weight participants recruited in the study. However, 6 participants from obese group were dropout (outlier data), thus their data was excluded (Fig. 1). Only 48 participants completed the study (obese = 18 and normal weight = 30) (Table 2). In terms of gender, both male and females were dominant among normal weight group. In respect to ethnicity, Malays were dominant among the obese group (Table 1). However, there was no significant difference in terms of gender and ethnicity between obese and normal weight groups. It is interesting to note that out of total 25 female participants 12 (66.7%) were obese; while out of 23 male participants 6 (33.3%) were obese (Table 1). Smoking status was significantly dominant among normal weight subjects (*p* = 0.021).

**Table 1:** Socio-demographic profile of the participants

Properties	Obese (*N* = 18)	Normal weight (*N* = 30)	*p* - value
Age (years) [Mean(± SE)]	44.7 (2.4)	47.2 (1.9)	0.405^a^
BMI [Mean(± SE)]	33.3 (0.9)	23.8 (0.5)	0.000^a*^
N (%)			
Gender			
Male	6 (33.3)	17 (56.7)	0.117^b^
Female	12 (66.7)	13 (43.3)	
Ethnicity			
Malay	13 (72.2)	12 (40.0)	0.067^b^
Chinese	1 (5.6)	8 (26.7)	
Indian	4 (22.2)	10 (33.3)	
Smoking habit			
Smoker	3 (16.7)	16 (53.3)	0.021^b*^
Non-smoker	14 (77.7)	11 (36.7)	
Former smoker	1 (5.6)	3 (10.0)	
Education level			
Tertiary	6 (33.3)	6 (20.0)	0.587^b^
Secondary	7 (38.9)	14 (46.7)	
Primary	5 (27.8)	10 (33.3)	
Disease status			
Diabetic	3 (16.7)	1 (3.3)	0.106^b^
Non-diabetic	15 (83.3)	29 (96.7)	

^a^Independent *T*-test; ^b^Chi-square test; ^*^Significant *p*-value < 0.05, Primary education level (Primary school), Secondary education level (Secondary school), Tertiary education level (College or university)

### Measurement of periodontal clinical parameters

Comparison of mean clinical parameters between both obese and normal weight group with periodontitis at baseline were showed at Table 2. At baseline, obese group showed higher VPI and GBI but lower PPD and CAL compared with normal weight group. Both the obese and normal weight groups were shown to have reduce percentages of VPI and GBI significantly at 12 weeks post NSPT (*p* <  0.5) (Table 2). It is important to note that obese group had significantly higher level (p <  0.5) of both VPI and GBI at the baseline compared to those of normal weight group. When comparisons were made between groups, the obese group experienced significantly higher mean reduction in VPI (69%) and GBI (15%) compared to the normal weight group (p <  0.5) (Table 2). Both obese and normal weight group showed significant reduction of mean PPD following 12 weeks NSPT. However, no mean changes different was reported when comparison were made between groups.

**Table 2:** Comparison of mean periodontal clinical parameters at baseline and 12 weeks following NSPT

Variables/Groups	Baseline	12 weeks after NSPT	Mean Reduction after NSPT
VPI (%)			
Obese	83.1 (72.3, 93.8)	13.8 (6.5, 21.0)^*^	69.3 (55.1, 83.4)^#^
Normal weight	45.5 (38.0, 53.0)	30.5 (24.1, 37.0)^*^	15.0 (9.2, 20.7)
GBI (%)			
Obese	79.1 (68.7, 89.6)	20.3 (12.5, 28.2)^*^	58.8 (45.6, 72.0)^#^
Normal weight	40.7 (35.5, 46.0)	25.9 (20.5, 31.2)^*^	14.9 (9.0, 20.7)
PPD (mm)			
Obese	2.4 (2.3, 2.6)	2.1 (1.9, 2.2)^*^	0.4 (0.2, 0.6)
Normal weight	3.0 (2.7, 3.2)	2.3 (2.1, 2.5)^*^	0.6 (0.5, 0.8)
CAL (mm)			
Obese	3.1 (2.8, 3.5)	2.7 (2.4, 3.0)	0.5 (0.3, 0.7)
Normal weight	3.7 (3.3, 4.1)	3.1 (2.7, 3.5)	0.6 (0.4, 0.7)

^*^Significant difference within group, ^**#**^Significant difference between group; mean differences were analyzed at 95% CI (/)

Further analysis was carried out using multiple regressions (Table 3) as summarized to find out whether obesity status remains the predictor for mean changes in VPI and GBI. After adjusting for smoking habit, obesity status was found significant as a predictor for clinical parameters of VPI and GBI with *p* < 0.05. Therefore, obese subjects would have 54.3 and 43.9% times higher mean change of VPI and GBI respectively compared to normal weight when anthropometric properties were similar.

**Table 3:** Multiple regression clinical parameters with subjects’ characteristic

Variables	Model	B	t	*p* - value
Mean changes				
VPI (%)	Obese	54.32	8.60	< 0.001^*^
GBI (%)	Obese	43.9	7.23	< 0.001^*^

Adjusted smoking habit to predict the changes of mean VPI and GBI

^*^Significant *p*–value < 0.05

### Serum Resistin and periodontal pathogens

The difference between means of serum resistin level at baseline and 12 weeks after NSPT of either obese or normal weight group were not statistically significant (Table 4). No significant changes were found for mean count for *P. gingivalis* (1.7 ×  10^6^ copy cells) and *T. forsythia* (0.6 × 10^6^ copy cells) at baseline and at 12 weeks post-NSPT in the obese group (Table 5). However, the mean count of *P. intermedia* had decreased [95% CI (0.7, 1.3; 0.4, 0.9)] to almost half after 12 weeks post NSPT. Mean counts for *P. gingivalis, T. forsythia*, and *P. intermedia* were reduced at 12 weeks post NSPT in the normal weight group.

**Table 4:** Comparisons of serum resistin within and between groups

Resistin (ng/mL)/Group	Time		Mean Change (95% CI)
	Baseline	12 weeks	
	Mean (95% CI)	Mean (95% CI)	
Resistin (ng/mL)			
Obese	14.7 (10.8, 18.5)	17.6 (12.4, 22.7)	2.9 (− 3.4, 9.3)
Normal weight	6.9 (4.3, 9.6)	9.5 (6.9, 12.0)	2.5 (0.9, 4.1)

Repeated measures ANOVA

**Table 5:** Comparisons mean counts of periodontal pathogens within and between groups

Periodontal pathogens/Groups (× 10^6^ copy cells)	Mean count (95% CI)	Mean count (95% CI)	Mean Change (95% CI)
*P. gingivalis*			
Obese	1.7 (1.5, 2.0)	1.5 (1.3, 1.7)	0.2 (0.1, 0.5)
Normal weight	1.8 (1.5, 2.0)	1.4 (1.1, 1.7)	0.4 (0.1, 0.7)
*T. forsythia*			
Obese	0.6 (0.4, 0.8)	0.7 (0.5, 0.9)	0.1 (− 0.1, 0.3)
Normal weight	0.9 (0.8, 1.0)	0.8 (0.6, 0.9)	0.1 (− 0.1, 0.3)
*P. intermedia*			
Obese	1.0 (0.7, 1.3)	0.6 (0.4, 0.9)	0.3 (0.1, 0.6)
Normal weight	1.1 (0.9, 1.2)	1.1 (1.0, 1.3)	0.1 (− 0.1, 0.3)

Repeated measures ANOVA

## Discussion

Periodontitis is a chronic inflammatory response that causes gingival inflammation and destruction of hard tissues [25]; with 30 to 35% prevalence in the general population worldwide [26]. Interestingly, the prevalence of periodontitis among obese populations worldwide has been reported between 47 to 74% [16, 27, 28]. Clinical conditions of periodontitis might aggravate in patients with other health complication such as obesity [29]. A positive but weak association between obesity and periodontitis has been reported [30], with unclear underlying biological mechanisms.

The present study reported no significant difference in mean serum resistin levels between baseline and 12 weeks after NSPT in both obese and normal weight groups. The current study findings contradicted other short-term studies, with a gap of 10–12 weeks to measure serum resistin following NSPT [31, 32]. Suresh and co-workers (2018) reported greater reduction in resistin which was observed in normal weight periodontitis than obese periodontitis at 8 weeks post-NSPT [9]. Even though short-term studies can demonstrate improvements of clinical parameters; however, it is not the same with serum level of cytokines and resistin. This notion is supported by Ide et al. (2003), who reported that short term study of 12 week duration could be insufficient to establish any biochemical changes following NSPT [32]. Thus, a study with a longer duration is recommended to observe meaningful changes in biochemical parameters. Tonetti and colleagues (2007) showed that systemic biomarkers were reduced at 6 months following NSPT [33]. Although these findings were observed at day 1 following NSPT; the levels of systemic biomarkers continued to be more regular after 6 months. D’Aiuto et al. (2005) in a systematic review suggested that NSPT could trigger a short term inflammatory response which was followed by a consistent progressive reduction in systemic inflammation and an improvement in endothelial function [34]. The obese individuals showed exaggerated inflammatory response [35]. However, a single-round of intensive NSPT could have triggered a short-term acute inflammatory response, resembling acute changes in the oxidative stress following NSPT [34]. A significant increase in diacron reactive oxygen metabolites (D-ROM) after NSPT has been reported, with a positive linear correlation between D-ROM levels and systemic inflammation biomarkers. The acute inflammatory response may have been triggered by the inevitable periodontal tissue damage during the procedure. Thus, the acute inflammatory findings in relation to oxidative stress could be explained by the increase in serum resistin in the obese group following NSPT in the present study.

In the current study, there were non-significant changes in mean count of *P. gingivalis*, *T. forsythia* and *P. intermedia* following NSPT in both groups (Table 5). It is to be noted that there is no report on the changes of periodontal pathogens count before and after NSPT in periodontitis with obesity. Previous studies showed that mean count of *P. gingivalis*, *T. forsythia* and *P. intermedia* could be reduced by 7–45% [36, 37] at 12 weeks post-NSPT in CP. However, the reductions of the similar bacterial flora were ranged between 18 to 99% after NSPT in periodontitis with diabetes [38, 39]. The findings from these studies were consistent with the present study in which NSPT is effective at reducing periodontal pathogens in normal weight subjects.

The study design was developed before 2017 Classification for Periodontal Disease and Peri-implant Disease and Conditions, thus the earlier case definition by Eke et al. (2012) was used [16]. At baseline, the obese group had almost double mean values of VPI and GBI, reflective of its inflammatory condition (Table 2). This concurs with current understanding that obese is a chronic inflammatory condition, which could contribute to an increase in the existing inflammatory burden associated with periodontitis [40]. This notion is further supported by Suvan et al., (2014) [41] who suggested obesity could have triggered greater inflammatory burden in other co–morbidities including periodontitis. This could imply that obesity condition may compromise the healing efficiency in those with periodontitis.

In addition, the obese group had lesser severity of periodontitis (lower means PPD and CAL) compared to normal weight group (Table 2). This could be explained by the fact that the normal weight group had significantly higher number of smokers (Table 1). It is well established that cigarette smoking remains an independent risk factor for development and progression of periodontitis, even after controlling for variables such as oral hygiene, plaque, calculus, socio-economic and demographical factors. Smoking has been associated with increasing host susceptibility towards periodontitis; as well as increasing periodontitis severity, by influencing the healing potential following treatment by means of its reduced angiogenesis nature. The risk associated was estimated with odd ratios of 2.5 to 6.0 [42–44]. It is worth mentioning that majority of the PPD were measured as less than 4 mm indicating a mild periodontitis among the participants of the study.

NSPT has been reported as an effective treatment to reduce local inflammatory burden [34]. In this study, the means of VPI, GBI and PPD were significantly reduced after NSPT. The findings were in line with other previous studies [18, 45]. It is important to note that, clinical parameters such as VPI, GBI and PPD are associated with active diseased state. Thus, it was expected that participants in both groups would have responded to NSPT (as a mean of managing active disease) by means of improvement in means VPI, GBI and PPD. However, NSPT did not result in significant reduction in mean CAL, and that could be explained by the fact that CAL accumulation is more influenced by the past disease experience rather than the current periodontitis status. CAL is also an estimate of accumulative periodontal tissue destruction [46, 47]. The extent of the disease would depend on how long the investigated population has been exposed to the disease.

When compared between groups, the obese group showed higher mean changes of VPI and GBI compared to the normal weight group, with statistical significant differences following NSPT (Table 2). This finding was contradictory to previous studies which reported reduction in the mean changes of VPI and GBI, but of no statistically significant difference [45]. Other study reported significant difference in mean change of CAL at 3 months following NSPT; however no significant difference in other parameters was identified [18]. The authors deduced that obesity does not contribute to the total burden of inflammation and suggested both obese and non-obese participants may have responded equally well to NSPT based on the intragroup results [18, 45].

We carried out linear (multiple) regression analysis (Table 3) to find out whether obesity could be a predictor for mean changes in VPI and GBI. After adjusting for smoking habit, obesity was found to be a significant predictor for VPI and GBI (*p* < 0.05). Obese participants were shown to have 54.3 and 43.9% time higher mean changes of VPI and GBI respectively compared to their normal weight counterpart with similar anthropometric profile. Clearly, NSPT is effective in reducing local inflammation in periodontitis obese and normal weight. However, cigarette smoking might have compromised the host healing potential resulting in relatively lower mean changes of VPI and GBI in the normal weight group following NSPT. It is worth to note that the majority of participants (Table 1) in this study were non diabetic, which is another risk factor for periodontitis.

Post hoc sample size calculation was carried out using G*Power software version 3.1 [19]. The calculation was based on detectable mean difference CAL between 2 groups obtained from this study. With a standard error of 5% (*p* = 0.05), it demonstrated the power of 43% (0.43). In view of medium mean difference CAL (0.71 mm) obtained from the study, the 18 obese and 30 normal weight should be acceptable. The results were deemed acceptable for the sample size used.

Limitations in this study include (i) most of the participant had mild periodontitis and (ii) short time monitoring. Future study should include involvement of obese with moderate to severe periodontis participants. Hopefully, this could be useful to evaluate the potential outcome of NSPT and bring about conclusive outcomes, especially to rule out the true effects of obesity and periodontis. At the same time, further consideration should be given on the length of the study period with increased frequency of monitioring the other clinical parameters.

## Conclusion

Regardless of obesity status, NSPT has a significant impact on VPI and GBI in periodontitis subjects. However, the impact of NSPT towards serum resistin and periodontal pathogens was non-significant in those with periodontitis.

## Data Availability

All data and materials of this work are available from the corresponding author on request.

## Abbreviations

BMI: Body mass index
CAL: Clinical attachment loss
ELISA: Enzyme-linked immune-sorbant assay
GBI: Gingival bleeding index
NSPT: Non-surgical periodontal therapy
PPD: Probing pocket depth
qPCR: Real-time quantitative polymerase chain reaction
VPI: Visible plaque index
WHO: World health organization

## References

[1] Zander HA, Polson AM, Heijl LC (1976). Goals of periodontal therapy*. J Periodontol.

[2] Slots J, Ashimoto A, Flynn MJ, Li G, Chen C (1995). Detection of putative periodontal in subgingival specimens by 16S ribosomal DNA amplification with the polymerase chain reaction. Clin Inf Dis.

[3] Eguchi T, Koshy G, Umeda M, Iwanami T, Suga J, Nomura Y (2008). Microbial changes in patients with acute periodontal abscess after treatment detected by PadoTest. Oral Dis.

[4] Asad M, Aziz AWA, Rahman RPC, Harun HAW, Ali TBT, Chinna K (2017). Comparison of nonsurgical periodontal therapy with oral hygiene instruction alone for chronic periodontitis. J Oral Sci.

[5] Akram Z, Baharuddin NA, Vaithilingam RD, Rahim ZHA, Chinna K, Krishna VG (2017). Effect of nonsurgical periodontal treatment on clinical periodontal variables and salivary resistin levels in obese Asians. J Oral Sci.

[6] Akram Z, Safii SH, Vaithilingam RD, Baharuddin NA, Javed F, Vohra F (2016). Efficacy of non-surgical periodontal therapy in the management of chronic periodontitis among obese and non-obese patients: a systematic review and meta-analysis. Clin Oral Inv.

[7] Kaushal S, Singh AK, Lal N, Das SK, Mahdi AA (2019). Effect of periodontal therapy on disease activity in patients of rheumatoid arthritis with chronic periodontitis. J Oral Biol Cran Res.

[8] Patel SP, Raju PA (2014). Gingival crevicular fluid and serum levels of resistin in obese and non-obese subjects with and without periodontitis and association with single nucleotide polymorphism at−420. J Indian Soc Periodontol.

[9] Suresh S, Mahendra J, Singh G, Kumar ARP, Thilagar S, Rao N (2018). Effect of nonsurgical periodontal therapy on plasma-reactive oxygen metabolite and gingival crevicular fluid resistin and serum resistin levels in obese and normal weight individuals with chronic periodontitis. J Indian Soc Periodontol.

[10] Goncalves TED, Feres M, Zimmermann GS, Faveri M, Figueiredo LC, Braga PG (2015). Effects of scaling and root planning on clinical response and serum levels of adipocytokines in patients with obesity and chronic periodontitis. J Periodontol.

[11] Suresh S, Mahendra J, Kumar A, Singh G, Jayaraman S, Paul R (2017). Comparative analysis of subgingival red complex bacteria in obese and normal weight subjects with and without chronic periodontitis. J Indian Soc Periodontol.

[12] Haffajee AD, Socransky SS (2009). Relation of body mass index, periodontitis and *Tannerella forsythia*. J Clin Periodontol.

[13] Filková M, Haluzík M, Gay S, Šenolt L (2009). The role of resistin as a regulator of inflammation: implications for various human pathologies. Clin Immunol.

[14] Steppan CM, Bailey ST, Bhat S, Brown EJ, Banerjee RR, Wright CM (2001). The hormone resistin links obesity to diabetes. Nature.

[15] Akram Z, Rahim ZHA, Ali TBT, Shahdan MSA, Baharuddin NA, Vaithilingam RD (2017). Resistin as a potential biomarker for chronic periodontitis: a systematic review and meta analysis. Arch Oral Biol.

[16] Eke PI, Dye BA, Wei L, Thornton-Evans GO, Genco RJ (2012). Prevalence of periodontitis in adults in the United States: 2009 and 2010. J Dent Res.

[17] WHO. Global database on body mass index; 2012.

[18] Altay U, Gurgan CA, Agbaht K (2013). Changes in inflammatory and metabolic parameters after periodontal treatment in patients with and without obesity. J Periodontol.

[19] Faul F, Erdfelder E, Lang AG, Buchner A (2007). G*power 3: a flexible statistical power analysis program for the social, behavioral, and biomedical sciences. Behavior Res Meth.

[20] Ainamo J, Bay I (1975). Problems and proposals for recording gingivitis and plaque. Int Dent J.

[21] Swierkot K, Nonnenmacher CI, Mutters R, Flores-de-Jacoby L, Mengel R (2009). One-stage full-mouth disinfection versus quadrant and full-mouth root planing. J Clin Periodontol.

[22] Raman RP, Taiyeb-Ali TB, Chan SP, Chinna K, Vaithilingam RD (2014). Effect of nonsurgical periodontal therapy verses oral hygiene instructions on type 2 diabetes subjects with chronic periodontitis: a randomised clinical trial. BMC Oral Health.

[23] Boutaga K, van Winkelhoff AJ, Vandenbroucke-Grauls CM, Savelkoul PH (2003). Comparison of real-time PCR and culture for detection of *Porphyromonas gingivalis* in subgingival plaque samples. J Clin Mic.

[24] Boutaga K, van Winkelhoff AJ, Vandenbroucke-Grauls CM, Savelkoul PH (2005). Periodontal pathogens: a quantitative comparison of anaerobic culture and real-time PCR. FEMS Imm Med Mic.

[25] Strohm TF, Alt KW. Periodontal disease—etiology, classification and diagnosis. In Den Anth. 1998:227–46.

[26] WHO. The WHO global oral health data bank. Geneva: World Health Organization; 2007.

[27] Khader YS, Bawadi HA, Haroun TF, Alomari M, Tayyem RF (2009). The association between periodontal disease and obesity among adults in Jordan. J Clin Periodonto.

[28] Khan S, Saub R, Vaithilingam RD, Safii SH, Vethakkan SR, Baharuddin NA (2015). Prevalence of chronic periodontitis in an obese population: a preliminary study. BMC Oral Health.

[29] Prentice AM, Jebb SA (2001). Beyond body mass index. Obes Rev.

[30] Chaffee BW, Weston SJ (2010). Association between chronic periodontal disease and obesity: a systematic review and meta-analysis. J Periodontol.

[31] Behle JH, Sedaghatfar MH, Demmer RT, Wolf DL, Celenti R, Kebschull M (2009). Heterogeneity of systemic inflammatory responses to periodontal therapy. J Clin Periodontol.

[32] Ide M, McPartlin D, Coward PY, Crook M, Lumb P, Wilson RF (2003). Effect of treatment of chronic periodontitis on levels of serum markers of acute-phase inflammatory and vascular responses. J Clin Periodontol.

[33] Tonetti MS, D’Aiuto F, Nibali L, Donald A, Storry C, Parkar M (2007). Treatment of periodontitis and endothelial function. New England J Med.

[34] D’Aiuto F, Parkar M, Tonetti MS (2005). Periodontal therapy: a novel acute inflammatory model. Inflam Res.

[35] Chapple IL, Matthews JB (2007). The role of reactive oxygen and antioxidant species in periodontal tissue destruction. Periodontol 2000.

[36] Ivić-Kardum M, Jurak I, Gall-Trošelj K, Pavelić K, Aurer A, Ibrahimagić L (2001). The effect of scaling and root planing on the clinical and microbiological parameters of periodontal diseases. Acta Stoma Croatica.

[37] Predin T, Djuric M, Nikolic N, Mirnic J, Gusic I, Petrovic D (2014). Clinical and microbiological effects of quadrant versus full-mouth root planning a randomized study. J Dent Sci.

[38] Christgau M, Palitzsch KD, Schmalz G, Kreiner U, Frenzel S (1998). Healing response to non-surgical periodontal therapy in patients with diabetes mellitus: clinical, microbiological, and immunologic results. J Clin Periodontol.

[39] Buzinin Samira Mukhtar, Alabsi Aied Mohammed, Tan Alexander Tong Boon, Vincent-Chong Vui King, Swaminathan Dasan (2014). Effects of Nonsurgical Periodontal Therapy on Clinical Response, Microbiological Profile, and Glycemic Control in Malaysian Subjects with Type 1 Diabetes. The Scientific World Journal.

[40] Genco RJ, Grossi SG, Ho A, Nishimura F, Murayama Y (2005). A proposed model linking inflammation to obesity, diabetes, and periodontal infections. J Periodontol.

[41] Suvan J, Petrie A, Moles DR, Nibali L, Patel K, Darbar U (2014). Body mass index as a predictive factor of periodontal therapy outcomes. J Dent Res.

[42] Bergström J (1989). Cigarette smoking as a risk factor in chronic periodontal disease. J Clin Periodontol.

[43] Bergström J (2004). Tobacco smoking and chronic destructive periodontal disease. Odontol.

[44] Bergström J, Preber H (1994). Tobacco use as a risk factor. J Periodontol.

[45] Zuza EP, Barroso EM, Carrareto ALV, Pires JR, Carlos IZ, Theodoro LH (2011). The role of obesity as a modifying factor in patients undergoing non-surgical periodontal therapy. J Periodontol.

[46] Haffajee AD, Socransky SS, Goodson JM (1983). Clinical parameters as predictors of destructive periodontal disease activity. J Clin Periodontol.

[47] Petersen PE, Ogawa H (2005). Strengthening the prevention of periodontal disease: the WHO approach. J Periodontol.

